# Loss of the R2R3 MYB Transcription Factor RsMYB1 Shapes Anthocyanin Biosynthesis and Accumulation in *Raphanus sativus*

**DOI:** 10.3390/ijms222010927

**Published:** 2021-10-10

**Authors:** Da-Hye Kim, Jundae Lee, JuHee Rhee, Jong-Yeol Lee, Sun-Hyung Lim

**Affiliations:** 1 Division of Horticultural Biotechnology, School of Biotechnology, Hankyong National University, Anseong 17579, Korea; kimdh143@naver.com; 2 National Institute of Agricultural Sciences, Rural Development Administration, Jeonju 54874, Korea; rheehk@korea.kr; 3 Department of Horticulture, Institute of Agricultural Science & Technology, Jeonbuk National University, Jeonju 54896, Korea; ajfall@jbnu.ac.kr

**Keywords:** anthocyanin, frameshift mutation, MBW complex, radish, RsMYB1

## Abstract

The red or purple color of radish (*Raphanus sativus* L.) taproots is due to anthocyanins, which have nutritional and aesthetic value, as well as antioxidant properties. Moreover, the varied patterns and levels of anthocyanin accumulation in radish roots make them an interesting system for studying the transcriptional regulation of anthocyanin biosynthesis. The R2R3 MYB transcription factor RsMYB1 is a key positive regulator of anthocyanin biosynthesis in radish. Here, we isolated an allele of *RsMYB1*, named *RsMYB1^Short^*, in radish cultivars with white taproots. The *RsMYB1^Short^* allele carried a 4 bp insertion in the first exon causing a frame-shift mutation of RsMYB1, generating a truncated protein with only a partial R2 domain at the N-terminus. Unlike RsMYB1^Full^, RsMYB1^Short^ was localized to the nucleus and the cytoplasm and failed to interact with their cognate partner RsTT8. Transient expression of genomic or cDNA sequences for *RsMYB1^Short^* in radish cotyledons failed to induce anthocyanin accumulation, but that for *RsMYB1^Full^* activated it. Additionally, *RsMYB1^Short^* showed the lost ability to induce pigment accumulation and to enhance the transcript level of anthocyanin biosynthetic genes, while *RsMYB1^Full^* promoted both processes when co-expressed with *RsTT8* in tobacco leaves. As the result of the transient assay, co-expressing *RsTT8* and *RsMYB1^Full^*, but not *RsMYB1^Short^*, also enhanced the promoter activity of *RsCHS* and *RsDFR*. We designed a molecular marker for *RsMYB1* genotyping, and revealed that the *RsMYB1^Short^* allele is common in white radish cultivars, underscoring the importance of variation at the RsMYB1 locus in anthocyanin biosynthesis in the radish taproot. Together, these results indicate that the nonsense mutation of RsMYB1 generated the truncated protein, RsMYB1^Short^, that had the loss of ability to regulate anthocyanin biosynthesis. Our findings highlight that the frame shift mutation of RsMYB1 plays a key role in anthocyanin biosynthesis in the radish taproot.

## 1. Introduction

Anthocyanins are flavonoid-derived metabolites with multiple functions, which include attracting pollinators or seed dispersal agents, protecting plants against damage from UV radiation, and contributing to cold and drought stress responses [1,2]. In addition, anthocyanins have strong antioxidant activity and have attracted widespread interest due to their health benefits in preventing chronic human diseases, certain cancers, and cardiovascular diseases [3,4,5].

The anthocyanin biosynthetic pathway has been well characterized and genes encoding the relevant enzymes and transcriptional regulators have been identified in many plant species [6]. Anthocyanin biosynthesis is transcriptionally regulated by R2R3 MYB transcription factors (TFs), basic helix-loop-helix (bHLH) TFs, and WD40 repeat proteins, which interact to form MYB–bHLH–WD40 (MBW) complexes [7,8]. Among these regulators, the MYB proteins contain two conserved imperfect repeats (named R2 and R3) in their N terminus and a variable region in the C terminus, which is responsible for their regulatory activity [9,10]. The bHLH factors form transcriptional complexes at the promoters of anthocyanin biosynthetic genes by interacting with the R3 region of their R2R3 MYB partners [11,12]. Within the complex, the WD40 partners act as a docking platform and stabilize the interaction between the MYB and bHLH TFs, rather than exerting a direct regulatory function [7,13].

Different MYB genes are expressed at different levels in different tissues as a function of developmental stage and environmental conditions; the set of MYB TFs in a specific tissue conditions the differential accumulation of flavonoids in that tissue by affecting the target gene specificity of the MBW complex, and thus the expression of specific anthocyanin biosynthesis genes [14,15]. Therefore, the correct distribution of anthocyanins in plant tissues requires accurate spatial and temporal regulation of the flavonoid biosynthetic pathway and reflects tissue-specific combinations of transcription factors.

In the MBW complex, the R2R3 MYB determines the spatio-temporal pattern of anthocyanin production and accumulation [16,17]. Several studies have demonstrated that loss of R2R3 MYB function is accompanied by defects in anthocyanin accumulation [18,19]. For example, mutations in *FvMYB10-1* from diploid strawberry (*Fragaria vesca*) and *CgRuby1^short^* from citrus *(Citrus grandis*) impair the function of the encoded R2R3 MYB TFs in activating anthocyanin biosynthesis [18,19]. The insertions of long terminal repeat (LTR) transposable elements in an exon of octoploid strawberry (*F.* × *ananassa*) *FaMYB10-1* or an intron of Chinese cabbage (*Brassica rapa* L.) *BrMYB2* are similarly associated with the lack of pigmentation of these species [19,20]. Likewise, a mutation affecting the splicing of tomato (*Solanum lycopersicum*) *ANTHOCYANIN 2-like* (*SlAN2-like*) transcripts results in a complete loss of anthocyanin accumulation [21].

In some cases, the *cis*-regulatory element recognized by the R2R3 MYB may also be present in the promoter region of its encoding gene, thus forming an auto-regulatory loop. For example, comparison of the proximal *MdMYB10* promoter regions between white-fleshed and green-foliaged apples (*Malus domestica*) revealed a single copy of a 23 bp sequence, whereas red-fleshed and red-foliaged apples contained six copies of an almost identical sequence as a minisatellite-like structure that was critical to the modulation of *MdMYB10* transactivation [22]. Indeed, mutating or deleting this repeat sequence in the *MdMYB10* promoter caused a strong reduction in transactivation by preventing autoregulation. In addition, blood orange (*Citrus* × *sinensis* ‘Blood orange’) gained its intense coloration from an LTR-retrotransposon insertion in the promoter of a gene encoding an R2R3 MYB TF, leading to the transcriptional activation of *CgRuby1* [23]. Similarly, purple-fruited peppers (*Capsicum annuum*) harbor a non-LTR retrotransposon insertion in the *CaAN2* promoter that is sufficient to activate the transcription of *CaAN2* [24]. The red color displayed by red-fleshed strawberries is also strongly associated with enhanced expression of *FaMYB10-2* due to an insertion of a CACTA-like transposon (FaEnSpm-2) in its promoter [19].

Radish (*Raphanus sativus* L.) is a member of the Brassicaceae family, and an economically important taproot vegetable crop grown globally. Radish taproots vary in color from white to red, purple-pink, or green, or even display two colors, depending on the accumulation and distribution of anthocyanins and chlorophyll [25,26]. Therefore, radish provides an interesting system for the study of the regulation of anthocyanin biosynthesis. Moreover, genes involved in anthocyanin biosynthesis have been cloned in radish. For example, the radish *RsMYB1* and *TRANSPARENT TESTA 8* (*RsTT8*) genes encode an R2R3 MYB and a bHLH TF, respectively [12,27]. Transient co-infiltration of constructs expressing these two genes in tobacco (*Nicotiana tabacum*) leaves confirmed their contribution to anthocyanin accumulation and the activation of anthocyanin biosynthetic genes, including *CHALCONE SYNTHASE* (*RsCHS*) and *DIHYDROFLAVONOL 4-REDUCTASE* (*RsDFR*) [12].

The transcriptional and epigenetic regulation of the key activator of anthocyanin biosynthesis *RsMYB1* (also called *RsMYB90*) orchestrates anthocyanin biosynthesis in radish taproots [27,28,29]. For example, hypermethylation of the *RsMYB1* promoter results in low *RsMYB1* transcription rates and white-fleshed taproots due to the lack of anthocyanin biosynthesis [30]. In addition, a retrotransposon insertion into a gene encoding a flavonoid 3′-hydroxylase (F3′H) in radishes with purple taproots causes a red color due to pelargonidin-based anthocyanin accumulation [31].

Here, we identify a novel allele of *RsMYB1* (*RsMYB1^Short^*) and examine RsMYB1 function in determining anthocyanin accumulation in radish taproots. The variants *RsMYB1^Full^* from red/purple radish and *RsMYB1^Short^* from white radish confer their characteristic taproot colors by exerting opposite effects on the regulation of anthocyanin biosynthesis. Based on *RsMYB1* variants in various radish cultivars, we also developed a molecular marker for the marker-assisted selection and prediction of radish lines with red/purple taproots. This study thus helps unravel the regulatory mechanisms underlying anthocyanin biosynthesis in radish taproots.

## 2. Results

### 2.1. Anthocyanin Accumulation Determines the Red and Purple Color of Radish Roots

**Figure 1:**
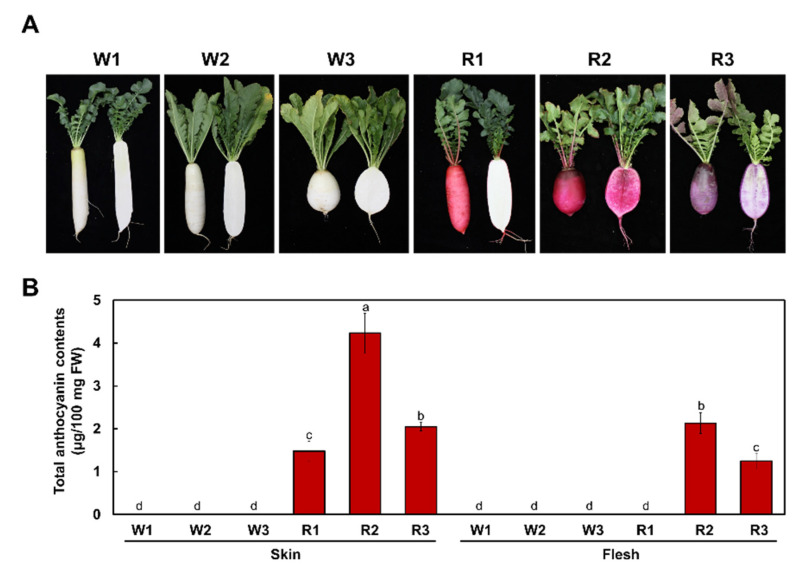
Phenotypes and anthocyanin contents of the six radish cultivars used in this study. (**A**) Representative photographs of white- and red-taproot radishes at the mature stage, with whole taproots on the left and taproots sectioned to show flesh color on the right of each image. (**B**) Anthocyanin levels in white and red taproot radishes. Results are mean values ± SD from three independent biological replicates. Different letters above the bars indicate significantly different values (*p* < 0.0001, one-way ANOVA followed by Duncan’s multiple range test).

We grew plants from all radish cultivars in a growth room and in the field to confirm their phenotypes after 8 weeks of growth. The three white cultivars W1, W2, and W3 showed no evidence of red color in their roots, whereas the skin of R1 cultivar taproots, as well as the root skin and root flesh of the R2 cultivar, had a clearly visible red color (Figure 1A). Interestingly, the R3 cultivar had purple root skin and flesh.

To quantify the pigment contents of root skins and flesh, we extracted anthocyanins from these tissues and measured their absorbance at 530 nm and 657 nm (Figure 1B). In agreement with the visible phenotypes above, we detected no anthocyanin accumulation in the root skins or flesh of the three white cultivars W1, W2, and W3. However, we measured high levels of anthocyanins in the root skin, but not in the flesh, of the R1 cultivar. Both the R2 and R3 cultivars accumulated anthocyanins in their root skins and flesh, with higher levels in the skin. Therefore, these results indicate that the pigmentation phenotype of radish taproots was determined by anthocyanin accumulation.

### 2.2. Anthocyanin Biosynthetic Genes Are Highly Expressed in Red and Purple Radish Taproots

**Figure 2:**
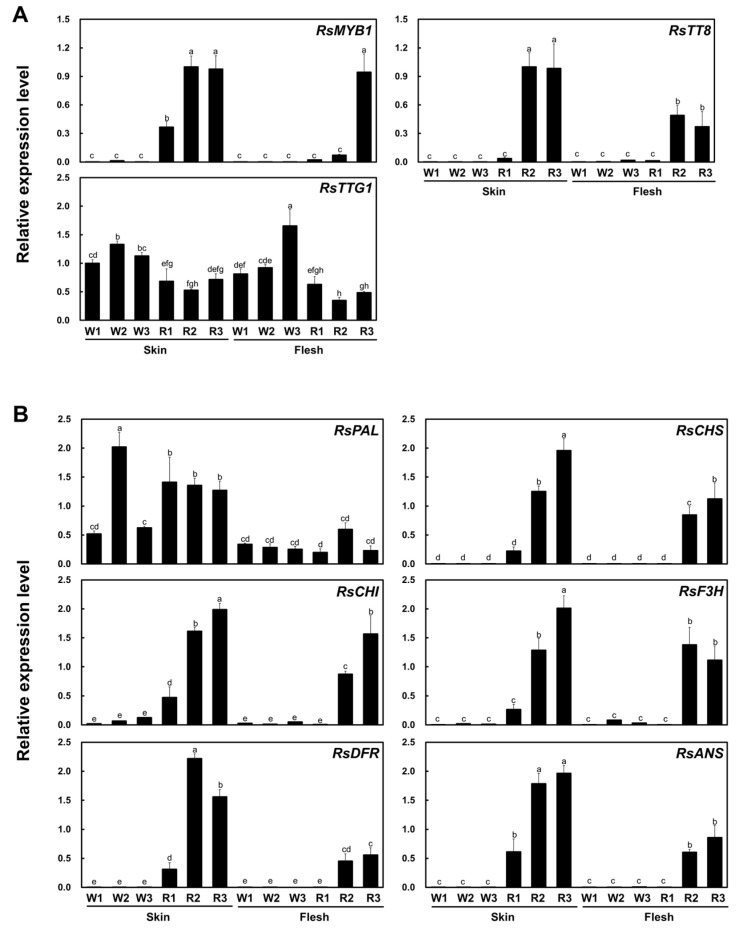
Relative transcript levels of anthocyanin biosynthetic and regulatory genes in the six radish cultivars. (**A**) Anthocyanin biosynthetic regulators. (**B**) Anthocyanin biosynthetic genes. *RsRPII* was used as a reference gene for evaluating the target gene expression. Results are mean values ± SD from three independent biological replicates. Different letters above the bars indicate significantly different values (*p* < 0.05, two-way ANOVA followed by Duncan’s multiple range test).

We next performed RT-qPCR on three previously identified regulators of anthocyanin biosynthesis, *RsMYB1*, *RsTT8*, and *TRANSPARENT TESTA GLABRA 1* (*RsTTG1*), in the root skin and flesh of the six radish cultivars. *RsMYB1* and *RsTT8* were highly expressed in the root skin of all red and purple cultivars, and their expression reflected the extent of pigment accumulation in these tissues (Figure 2A). In addition, *RsMYB1* and *RsTT8* were more highly expressed in the root skin than in the root flesh of colored cultivars. By contrast, and as previously reported, *RsTTG1* was expressed at similar levels in all tissues independently of anthocyanin accumulation. These results indicate that simultaneous expression of *RsMYB1* and *RsTT8* correlated with anthocyanin accumulation. Indeed, anthocyanin biosynthetic genes were generally expressed to much higher levels in tissues accumulating anthocyanins than in tissues from white radish cultivars (Figure 2B). The general phenylpropanoid biosynthetic gene, *PHENYLALANINE AMMONIA-LYASE* (*RsPAL*), was equally expressed between red and white cultivars. However, early biosynthetic genes (EBGs), including *RsCHS*, *CHALCONE ISOMERASE* (*RsCHI*), and *FLAVANONE 3-HYDROXYLASE* (*RsF3H*), were highly upregulated in the red and purple cultivars, which also show high expression of *RsMYB1* and *RsTT8*. Likewise, the transcript levels of the late biosynthetic genes (LBGs) *RsDFR* and *ANTHOCYANIDIN REDUCTASE* (*RsANS)* were high in the root skins and root flesh of the red and purple cultivars, in agreement with the expression pattern of *RsMYB1* and *RsTT8*. Taken together, these results confirm that anthocyanin accumulation reflected the expression levels of flavonoid biosynthetic genes across the different cultivars, and that this occurred in tissues co-expressing *RsMYB1* and *RsTT8*.

### 2.3. White Taproot Radish Cultivars Harbor a Variant RsMYB1 Allele

**Figure 3:**
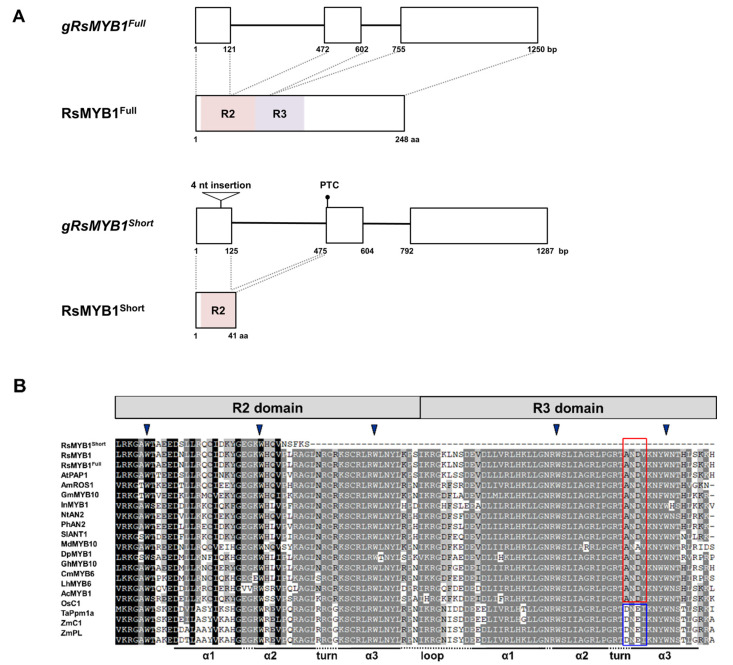
Genomic structure of *RsMYB1* alleles and phylogenetic tree of RsMYB1 proteins from red and white radish cultivars and R2R3 MYB proteins from other plants. (**A**) Schematic diagram of the genomic structure of *RsMYB1*. The predicted protein encoded by each mature mRNA is indicated below with their functional domains. White boxes, exons; black lines, introns. The 4 bp insertion site in *RsMYB1^Short^* is indicated by a triangle. Premature termination codon (PTC) is indicated by a circle. (**B**) Multiple protein sequence alignment of the R2 and R3 domains across R2R3 MYB proteins from other plants. The conserved residues of the DNEI and ANDV motifs are shown in the red and blue boxes, respectively. Inverted blue triangles indicate the conserved residues forming the inner hydrophobic core of the R2 and R3 domains.

To investigate the mechanism underlying the variation in taproot color, we compared the coding regions of *RsMYB1* and *RsTT8* from white (W1) and red (R1) radish cultivars. *RsMYB1* from the red cultivar exhibited a 93.0% sequence identity to the previously reported *RsMYB1* allele from the purple radish cultivar ‘Bordeaux’, due to the presence of an insertion/deletion (InDel) polymorphism in an intron and several single-nucleotide polymorphisms (SNPs) in both exons and introns (Supplementary Figure S1). The *RsMYB1* coding sequence from red radish (R1) was 747 bp in length and encoded a predicted protein of 248 amino acids, which we designated RsMYB1^Full^ (Figure 3A). Likewise, the sequence identity between *RsMYB1* from the white and Bordeaux cultivars was 94.5%, owing to an InDel in an intron and several SNPs in exons and introns. Notably, RsMYB1 from the white radish cultivar W1 contained a 4 bp (AATT) insertion in the first exon, resulting in the frameshift mutation and early stop codon after five amino acids from this mutation point (Figure 3B). The corresponding protein retained only the 41-amino-acid N-terminal part of RsMYB1^Full^ that lacked the majority of the downstream residues of the R2 domain and was designated as RsMYB1^Short^.

Sequence alignments between anthocyanin-promoting R2R3 MYB TFs belonging to the phylogenetic subgroup 5 (SG5) and subgroup 6 (SG6) from various plants indicated that RsMYB1^Full^ and RsMYB1^Short^ cluster with the SG6 clade (Supplementary Figure S2). In addition, this alignment showed that RsMYB1^Full^ has a typical R2R3 domain with a conserved ANDV motif and a domain through which such R2R3 TFs interact with bHLH TFs ([D/E]Lx2[R/K]x3Lx6Lx3R) in its N terminus, as well as the common SG6 (KPRPR[S/T]F) motif in its C terminus. In contrast, RsMYB1^Short^ had the only partial R2 domain and was largely truncated in the subsequent region, harboring the R3 domain and activation motif at the C terminus.

### 2.4. Subcellular Localization Analysis of RsMYB1^Short^, RsMYB1^Full^, and RsTT8

**Figure 4:**
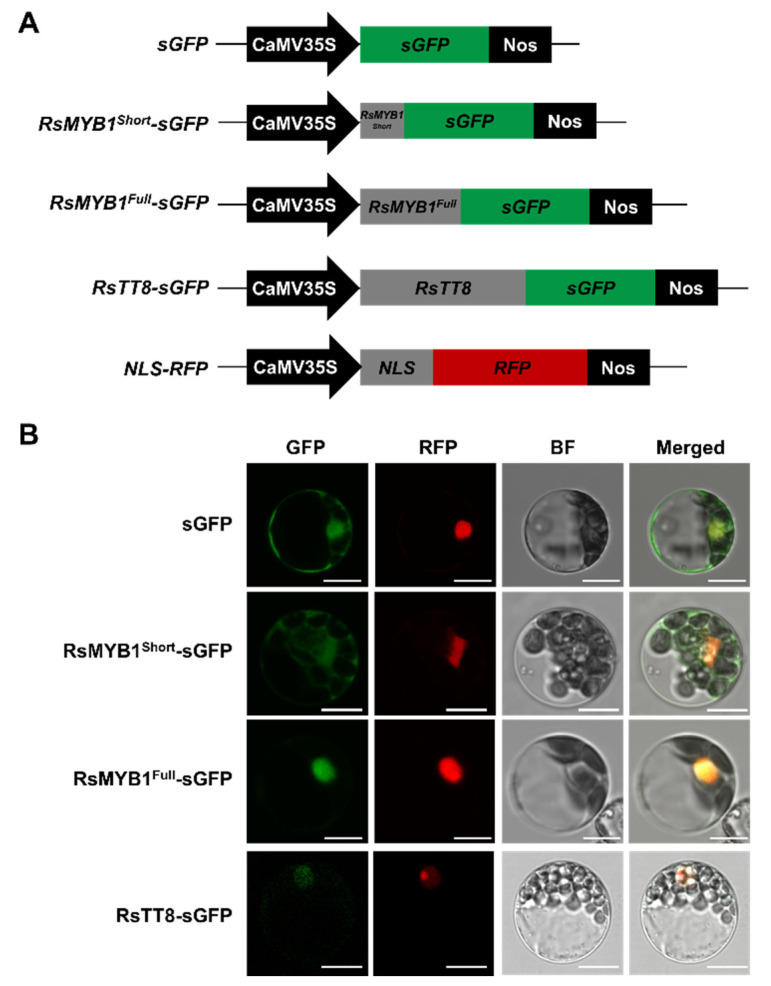
Subcellular localization of RsMYB1^Short^, RsMYB1^Full^, and RsTT8 in Arabidopsis leaf protoplasts. (**A**) Schematic diagrams of the constructs: CaMV35S, cauliflower mosaic virus *35S* promoter; *sGFP*, soluble green fluorescent protein (*GFP*) gene; *RsMYB1^Short^-sGFP*, *RsMYB1^Short^* fused to *sGFP*; *RsMYB1^Full^-sGFP*, *RsMYB1^Full^* fused to *sGFP*; *RsTT8-sGFP*, *RsTT8* fused to *sGFP*; *NLS-RFP*, nuclear localization signal fused to the red fluorescent protein (*RFP*) gene; *Nos*, nopaline synthase terminator. (**B**) RsMYB1^Short^, RsMYB1^Full^, and RsTT8 localize to the nucleus in Arabidopsis protoplasts. Data are representative of protoplasts accumulating each fusion protein 16 h after transfection. Scale bars = 10 μm.

For assessment of the subcellular distribution of RsMYB1^Short^ and RsMYB1^Full^, we transiently transfected Arabidopsis mesophyll protoplasts with constructs expressing fusions between the green fluorescent protein (GFP) and RsMYB1^Short^, RsMYB1^Full^, or RsTT8, together with a nuclear localization marker, consisting of the red fluorescent protein (RFP) with the nuclear localization signal (NLS) of the SV40 large T antigen, as a positive control (Figure 4A). We detected strong GFP fluorescence from the nuclei of all protoplasts transfected with the *RsMYB1^Full^-GFP* and *RsTT8-GFP* constructs that co-localized with RFP fluorescence (Figure 4B). However, green fluorescence from *RsMYB1^Short^-GFP* was distributed throughout the cell, including the nucleus and the cytoplasm. Therefore, these results indicate that the frame shift of RsMYB1 can lead to its abnormal subcellular distribution.

### 2.5. Frame Shift of RsMYB1 Affects Its Interaction with RsTT8

**Figure 5:**
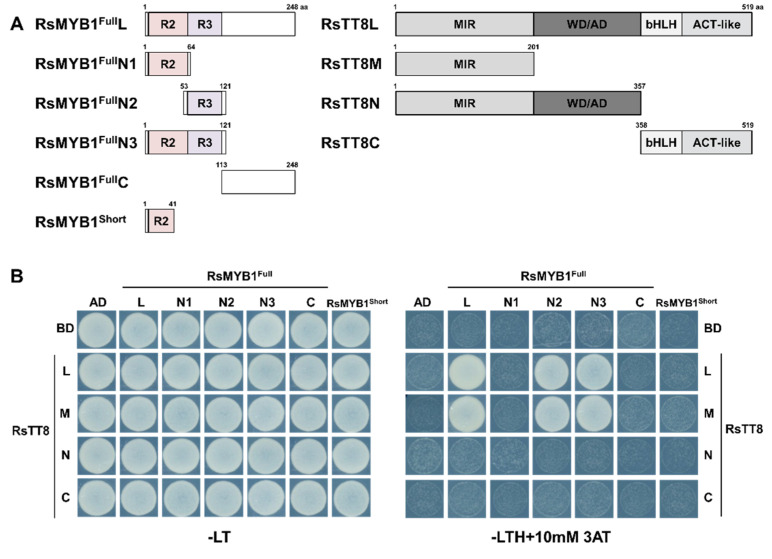
Physical interactions among RsMYB1^Full^, RsMYB1^Short^, and RsTT8. (**A**) Schematic diagrams of the constructs used in the Y2H assay. The amino acid positions of the fragments are shown. (**B**) Protein–protein interactions among RsMYB1^Full^, RsMYB1^Short^, and RsTT8, as revealed by Y2H analysis. SD/−LT, synthetic defined medium lacking Leu and Trp; SD/−LTH+3AT, SD medium lacking Leu, Trp, and His but containing 10 mM 3-amino-1,2,4-triazole (AT). AD and BD indicate the GAL4 activation domain and binding domain, respectively.

As shown in Figure 1, RsMYB1 and RsTT8 together regulate anthocyanin biosynthetic gene expression in skin and flesh of radish taproot. To examine the interaction between RsMYB1 and RsTT8, we constructed yeast two-hybrid constructs by placing full-length *RsMYB1^Short^* and full-length and partially truncated *RsMYB1^Full^* in-frame with the sequence encoding the GAL4 activation domain (GAL4-AD) from the pGADT7 vector (Figure 5A). We also generated a set of constructs in the pGBKT7 vector, placing full-length and truncated *RsTT8* in-frame with the sequence coding for the GAL4 DNA-binding domain (BD). We co-transformed appropriate pairs of AD and BD constructs into the yeast strain MaV203 and tested their interaction on stringent selective medium containing 10 mM 3-AT, a competitive inhibitor of the yeast HIS3 enzyme. These assays showed that RsMYB1^Full^ can interact with RsTT8, but RsMYB1^Short^ cannot. Indeed, we determined that RsTT8 interacts strongly with partially truncated RsMYB1^Full^N2, and RsMYB1^Full^N3 as well as RsMYB1^Full^L, but not with RsMYB1^Full^N1, which only harbored the R2 domain (Figure 5B). Notably, RsTT8M and RsTT8N, which contained the common MYB interaction region (MIR), interacted with RsMYB1^Full^N2, and RsMYB1^Full^N3, but not with RsMYB1^Full^N1 and RsMYB1^Short^. Taken together, these results indicate that the interaction between RsMYB1 and RsTT8 may be mediated by an intact R3 domain from RsMYB1 and the MIR of RsTT8, but does not require the bHLH domain of RsTT8.

### 2.6. RsMYB1^Short^ Is a Loss of Function Allele for Anthocyanin Biosynthesis

**Figure 6:**
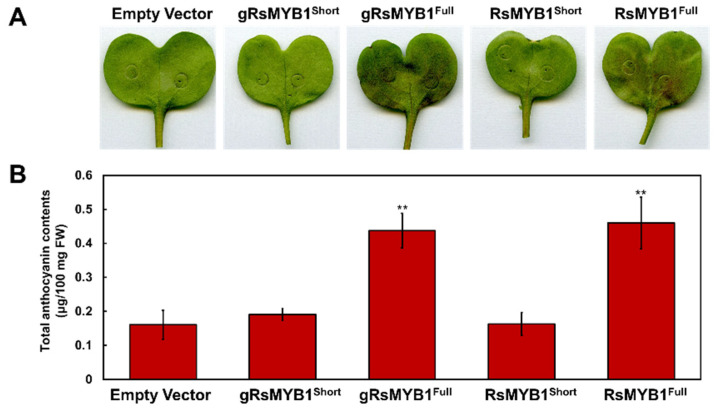
Functional analysis of RsMYB1^Short^ and RsMYB1^Full^ for anthocyanin biosynthesis. (**A**) Transient Agrobacterium-mediated infiltration assay of constructs expressing *RsMYB1* in radish cotyledons. (**B**) Anthocyanin contents in transiently infiltrated radish cotyledons shown in A. Results are mean values ± SD from three independent biological replicates. ** *p* < 0.01, as determined by Student’s paired *t*-test relative to the empty vector.

Given that RsMYB1^Short^ showed an abnormal subcellular distribution and deficient interacting ability with cognate RsTT8, we suspected that RsMYB1^Short^ might be lacking in transactivating its downstream targets. To test this hypothesis, we evaluated the function of RsMYB1^Short^ and RsMYB1^Full^ on anthocyanin biosynthesis via transient assay in radish cotyledons. The transient overexpression of the full length of the genomic sequence or the cDNA of *RsMYB1^Full^* induced visible pigment accumulation in radish cotyledons, showing that this protein activated anthocyanin biosynthesis. By contrast, neither genomic nor cDNA of *RsMYB1^Short^* activated anthocyanin biosynthesis, indicating that RsMYB1^Short^ was non-functional (Figure 6). These results thus suggest that the 4 bp insertion in *RsMYB1^Short^* was responsible for a dysfunctional protein that lost the RsMYB1 function.

**Figure 7:**
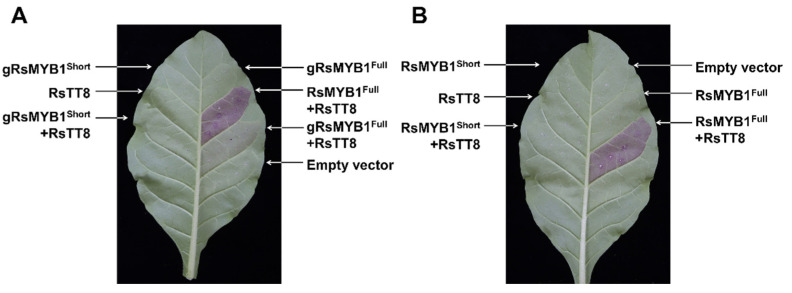

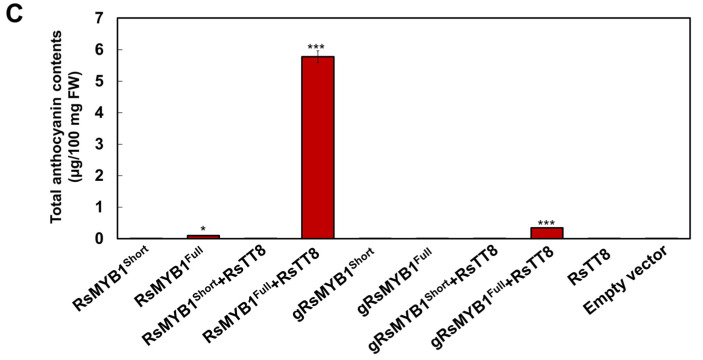
Visible phenotypes and anthocyanin contents of tobacco leaves transiently expressing *RsMYB1* constructs. (**A**) Tobacco leaves transiently infiltrated with constructs carrying the genomic sequence from *RsMYB1^Short^* or *RsMYB1^Full^* alone or together with *RsTT8*. (**B**) Tobacco leaves transiently infiltrated with constructs carrying the cDNAs from *RsMYB1^Short^* or *RsMYB1^Full^* alone or together with *RsTT8*. (**C**) Anthocyanin contents of the leaf sectors shown in A and B. Results are mean values ± SD from three independent biological replicates. * *p* < 0.05; and *** *p* < 0.001, as determined by Student’s paired *t*-test relative to the empty vector.

To obtain an independent confirmation of the respective functions of RsMYB1^Full^ and RsMYB1^Short^ in anthocyanin biosynthesis, we repeated the transient infiltration assay in tobacco leaves. Similar to the results with radish cotyledons, we detected the visible accumulation of pigments only with constructs expressing *RsMYB1^Full^* and *RsTT8*, as cDNAs or a genomic sequence (Figure 7A,B). However, the overexpression of *RsMYB1^Short^* did not induce pigment accumulation, with or without the co-expression of *RsTT8.* The extraction of anthocyanins from each leaf section corroborated the visible phenotypes (Figure 7C).

**Figure 8:**
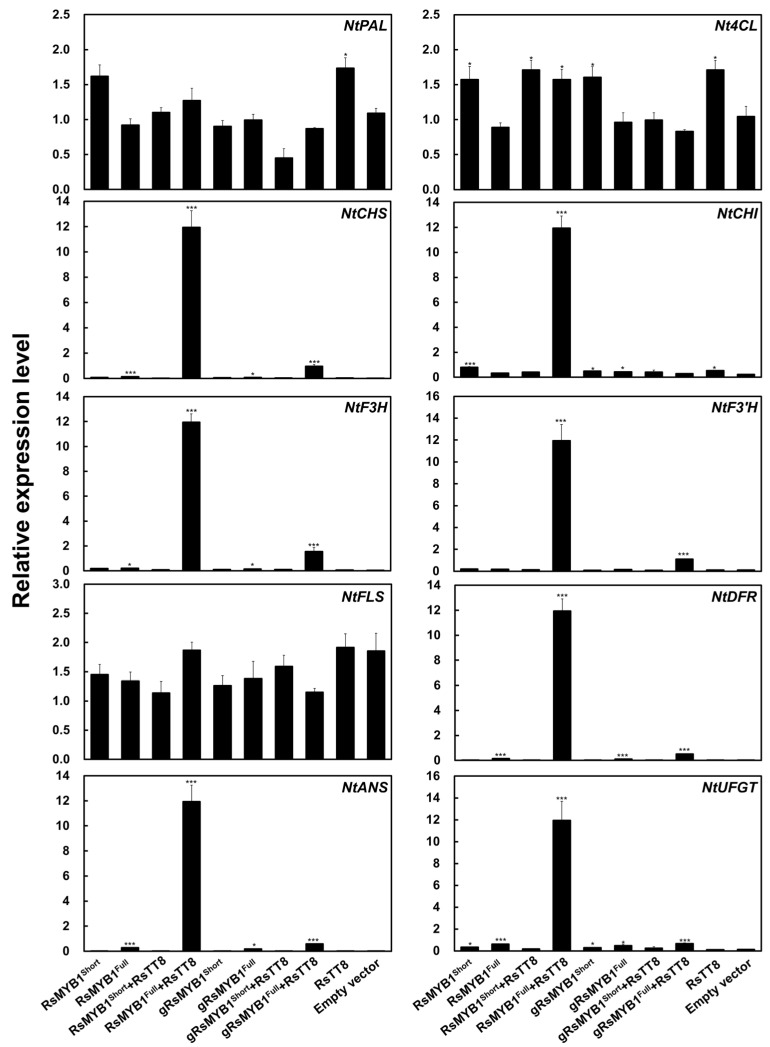
Relative transcript levels of anthocyanin biosynthetic genes in tobacco leaves transiently expressing *RsMYB1* constructs. *NtGAPDH* was used as a reference gene. Results are mean values ± SD from three independent biological replicates. * *p* < 0.05; *** *p* < 0.001, as determined by Student’s paired *t*-test relative to the empty vector.

To explore the relationship between the expression of anthocyanin biosynthesis genes and anthocyanin contents, we measured the transcript levels of ten structural genes involved in anthocyanin biosynthesis in infiltrated tobacco leaves (Figure 8). We focused on the upstream genes *NtPAL* and *4-COUMARATE:COA LIGASE* (*Nt4CL*), the EBGs *NtCHS*, *NtCHI*, *NtF3H*, *NtF3′H*, and *FLAVONOL SYNTHASE* (*NtFLS*), and the LBGs *NtDFR*, *NtANS*, and *UDP-GLUCOSE: FLAVONOID 3-O-GLUCOSYLTRANSFERASE* (*NtUFGT*). The transient overexpression of *RsMYB1^Short^* alone did not induce the transcription of any anthocyanin biosynthetic genes, as transcript levels were comparable to those of the control transfected with empty vector and to those following *RsTT8* transient overexpression. By contrast, transient expression of *RsMYB1^Full^* alone or in combination with *RsTT8* activated the transcription of anthocyanin biosynthetic genes, with the exception of *NtPAL*, *Nt4CL*, and *NtFLS*, which showed high transcript levels in all samples, including the control transfected with empty vector. We concluded from these results that the *RsMYB1^Full^* allele, but not the *RsMYB1^Short^* allele, was correctly transcribed and translated into a functional protein, even in a heterologous system such as tobacco. These results verify that the 4 bp insertion specific to *RsMYB1^Short^* led to a non-functional allele in both radish and tobacco.

### 2.7. RsMYB1^Short^ Cannot Transactivate the RsCHS and RsDFR Promoters

**Figure 9:**
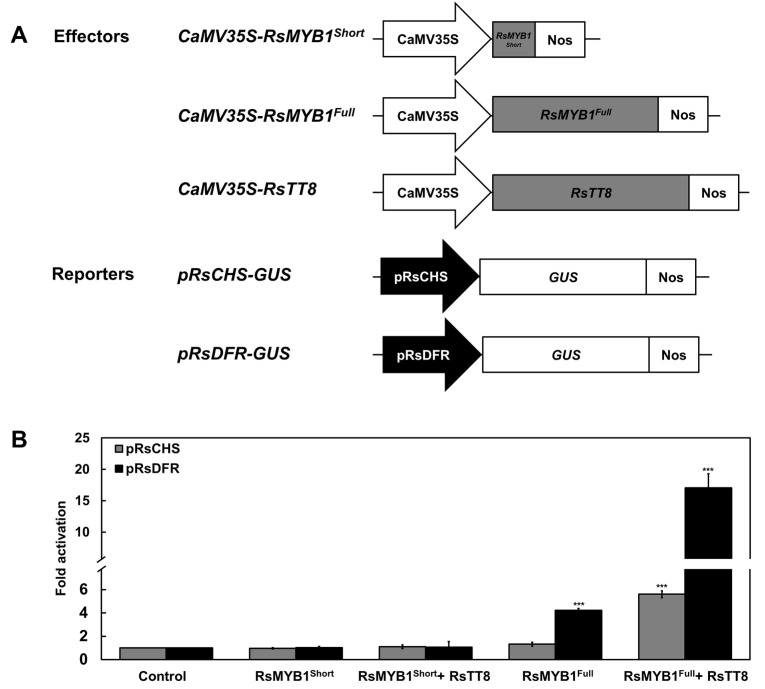
Transcriptional activation assay of the *RsCHS* and *RsDFR* promoters by RsMYB1^Full^, RsMYB1^Short^, and RsTT8. (**A**) Schematic diagrams of effector and reporter constructs used in the transcriptional activation assay. The effector construct harbors the coding sequences of *RsMYB1^Full^*, *RsMYB1^Short^*, and *RsTT8*, respectively, driven by the *35S* promoter and *NOS* terminator. The reporter constructs carry the *RsCHS* and *RsDFR* promoters driving *GUS* expression. (**B**) Regulatory consequences of the transient expression of *RsMYB1^Short^*, *RsMYB1^Full^*, and *RsTT8* on *RsCHS* and *RsDFR* promoter activity. Results are mean values ± SD from three independent biological replicates. *** *p* < 0.001, as determined by Student’s paired *t*-test relative to the control.

Previous studies reported that RsMYB1-mediated activation of the *RsCHS* and *RsDFR* promoters was enhanced by the RsTT8 [12]. To directly link RsMYB1^Short^ and RsMYB1^Full^ to anthocyanin biosynthesis, we performed a transient promoter activation assay in tobacco leaves by co-infiltrating effector constructs overexpressing the coding sequences of *RsMYB1^Short^*, *RsMYB1^Full^*, or *RsTT8* with reporter constructs in which the *ß-GLUCURONIDASE* (*GUS*) reporter gene was driven by the *RsCHS* or *RsDFR* promoter. As previously reported [12], overexpression of *RsMYB1^Full^* was sufficient to transactivate the *RsDFR* promoter, but not the *RsCHS* promoter (Figure 9). However, both the *RsCHS* and *RsDFR* promoters showed strong induction when the *RsMYB1^Full^* and *RsTT8* effector constructs were co-infiltrated. Notably, the overexpression of *RsMYB1^Short^* alone or with *RsTT8* failed to transactivate the *RsCHS* and *RsDFR* promoters. The Y2H assay demonstrated that RsMYB1^Short^ may exist in the small part of the R2 domain due to the frameshift mutation, resulting in the loss of the ability to interact with RsTT8 (Figure 5). These results indicate that the intactness of RsMYB1 plays a crucial role in anthocyanin biosynthesis, via interacting with its bHLH partner RsTT8.

### 2.8. Additional Unknown Factors Influence Anthocyanin Biosynthesis in Radish Taproots

**Figure 10:**
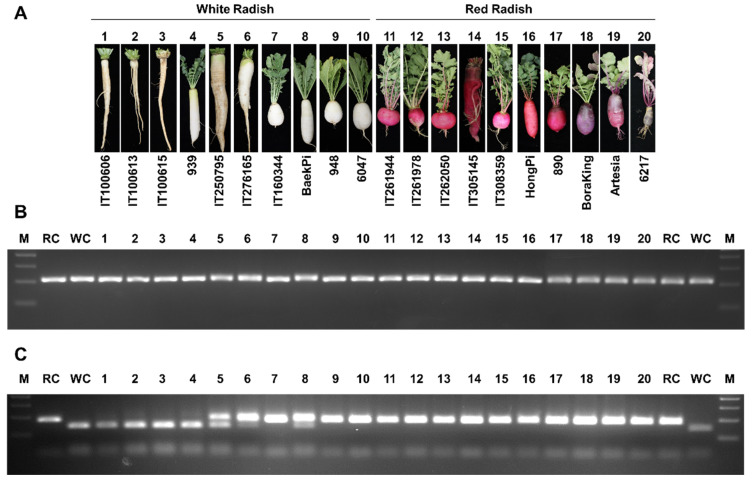
Sequence variation at *RsMYB1* predicts taproot color across 20 radish cultivars. (**A**) Representative photographs of radish taproots showing the different colors of the indicated cultivars. (**B**) Gel electrophoresis of amplified fragments of the RsMYB1 gene. (**C**) Genotyping results of the 20 radish cultivars with the CAPS marker for *RsMYB1*. FC and SC are PCR amplicons from plasmids harboring *RsMYB1^Short^* or *RsMYB1^Full^*, respectively, as positive controls. M: 100 bp DNA size marker.

With the goal of developing a molecular marker to discriminate between the *RsMYB1^Short^* and *RsMYB1^Full^* alleles and assess the association between the genotype at *RsMYB1* and taproot color, we analyzed 20 radish cultivars for variation at *RsMYB1*. Accordingly, we developed a cleaved amplified polymorphic sequence (CAPS) marker based on the variation in the first exon of *RsMYB1* (Supplementary Figure S1). We then genotyped 20 radish cultivars with different taproot colors using this new marker; as positive controls, we included PCR samples using plasmids containing genomic sequences from *RsMYB1^Short^* and *RsMYB1^Full^* as templates. Undigested PCR amplicons for *RsMYB1^Full^* and *RsMYB1^Short^* were 216 bp and 220 bp in size, respectively, but the 4 bp insertion specific to *RsMYB1^Short^* introduced a restriction site for the enzyme *Mlu*CI (Figure 10). Digested PCR products for the *RsMYB1^Full^* allele yielded two fragments (185 and 31 bp), while digested PCR products derived from the *RsMYB1^Short^* allele yielded three fragments (145, 44, and 31 bp), demonstrating the validity of the CAPS marker. Importantly, all radish cultivars with red taproots shared the *RsMYB1^Full^* allele, whereas cultivars with white taproots were either homozygous for the *RsMYB1^Short^* (four cultivars) or the *RsMYB1^Full^* allele (three cultivars), or heterozygous for *RsMYB1^Short^* and *RsMYB1^Full^* (three cultivars). These results indicate that the genotype at *RsMYB1* contributes to radish taproot color development. However, since the *RsMYB1* gene alone cannot distinguish radish taproot color, further studies on additional candidate genes for marker development are needed.

## 3. Discussion

We discovered that a 4 bp insertion of the anthocyanin biosynthesis regulator gene *RsMYB1* abolishes anthocyanin accumulation in radish. This insight into the regulatory mechanisms behind anthocyanin biosynthesis and its spatio-temporal control has significant implications for the development of designer plant and fruit varieties engineered to have higher or lower levels of anthocyanins through conventional or advanced breeding methods.

### 3.1. Spatial Expression of RsMYB1 and RsTT8 Affects Anthocyanin Accumulation in Radish Taproot

The tissue-specific expression of genes encoding MYB or bHLH TFs has been shown to control the site and timing of anthocyanin biosynthesis [32,33]. We confirmed that *RsMYB1* and *RsTT8* transcript levels correlated well with the extent of anthocyanin accumulation in the skin and flesh of taproots, as well as with transcript levels of early (EBG) and late (LBG) anthocyanin biosynthetic genes (Figure 2).

Importantly, we found that radish cultivars with white taproots produce *RsMYB1* transcripts that are predicted to encode a shorter protein, RsMYB1^Short^, with the 4 bp insertion in the first exon, causing a premature stop codon that terminates the protein at only about one-fifth of its functional length (Figure 3 and Supplementary Figure S1). We identified this insertion only in radish cultivars with white taproots, but not in those with red taproots, although some white-taproot cultivars were heterozygous for the *RsMYB1^Short^* and *RsMYB1^Full^* alleles (Figure 10).

In view of previous studies reporting that anthocyanin biosynthesis requires the interaction of RsMYB1 and RsTT8 [12], we investigated the molecular consequences of RsMYB1^Short^ on protein–protein interactions, and found that RsTT8 interacted with only RsMYB1^Full^, not RsMYB1^Short^. We also refined the interaction interface between RsMYB1 and RsTT8, showing the R3 domain of RsMYB1 was an indispensable region with the MIR at the N terminus of RsTT8. Indeed, the truncated version of RsMYB1, RsMYB1^Short^, lacking the R3 domain failed to interact with either full-length or partially truncated versions of its cognate partner, RsTT8. We confirmed that RsMYB1^Short^ was unable to transactivate the *RsCHS* and *RsDFR* promoters and did not result in anthocyanin accumulation when transiently overexpressed in radish cotyledons or tobacco leaves, expected by the loss of ability to interact with RsTT8. These results demonstrate that the interaction of RsMYB1 and RsTT8 can transcriptionally activate the anthocyanin biosynthetic genes and lead to anthocyanin accumulation.

### 3.2. The Allelic Variant RsMYB1^Short^ Lack of Function in Anthocyanin Biosynthesis

R2R3 MYB TFs have a highly conserved DNA-binding domain at their N terminus and a highly variable region at their C terminus, harboring the activation or repression motif that contributes to their functional grouping [34]. At the N terminus, each R2 and R3 domain consists of an imperfect repeat (R) of about 52 amino acids in length and forms three α-helices, resulting in a helix-turn-helix protein architecture that conforms to the major groove of genomic DNA [30]. Several studies reported that the R2 and R3 domains play an important role in the recognition of the DNA backbone, as well as the interaction with counterpart bHLH TFs. For example, in white-flowered lily (*Lilium speciosum*), a single amino acid substitution at a conserved aromatic residue (Trp to Leu) in the R2 repeat of the R2R3 MYB TF LsMYB12 completely suppresses anthocyanin biosynthesis [35]. Additionally, the transcript levels of *LsMYB12^WtoL^* are very low and lead to reduced transcription of anthocyanin biosynthetic genes, resulting in the absence of pigmentation in tepals. In tomato *SlAN2like^WT^*, alternative splicing of *R2R3 MYB SlAN2-like* led to the formation of the dysfunctional protein with only an R2 domain, which showed the complete loss of anthocyanin accumulation in fruit peel [21]. Additionally, SlAN2like^WT^ lost the ability to interact with bHLH partners, indicating that the formation of the MBW complex was not affected.

We demonstrated here that the *RsMYB1^Short^* transcripts encode truncated proteins with the loss of most of the downstream residue of the R2 domain. The R3 domain of R2R3 MYB TFs, orchestrating anthocyanin accumulation, is important for their interaction with the MIR of flavonoid-related bHLH TFs. This interaction is a prerequisite for the assembly of the anthocyanin-activating MBW complex [34]. The protein encoded by the allelic variant RsMYB1^Short^ exhibited abnormal subcellular distribution and lost the ability to physically interact with RsTT8 (Figure 4 and Figure 5). As expected, the simultaneous transient co-overexpression of *RsMYB1^Short^* and *RsTT8* did not activate the transcription of anthocyanin biosynthetic genes or result in anthocyanin accumulation. These results suggest that RsMYB1^Short^, a truncated protein with a partial R2 domain, should abolish the transcriptional regulation of anthocyanin biosynthetic genes and interfere with the formation of the MBW complex.

Another possible explanation is that the aberrant *RsMYB1^Short^* might affect the transcript levels of *RsTT8*, thus decreasing RsTT8 accumulation and interfering with formation of the MBW complex. Although the *RsTT8* locus in the white and red cultivars encoded the same protein, *RsTT8* transcript levels were very low in the taproot of the white cultivars tested here. Indeed, many studies have shown that R2R3 MYB enhances the transcription of the genes encoding the bHLH component of the MBW complex [36,37,38]. The Arabidopsis R2R3 MYB TFs’ production of anthocyanin pigment 1 (AtPAP1) and AtTT2 and tomato SlAN4 can increase the transcription of the bHLH-encoding genes *AtTT8* and *SlAN1*, respectively [36,38]. Taken together, these results suggest that the dysfunctional R2R3 MYB proteins interfere with the conformation of the active MBW complex by reducing the transcription of bHLH TF-encoding genes.

Self-regulation of *RsMYB1* by RsMYB1 may also contribute to the lack of anthocyanin biosynthesis in white radishes. Previous studies reported that the transcription of *MdMYB10* in red-fleshed apples is self-regulated by MdMYB10 binding to its promoter [22]. As RsMYB1^Short^ only harbors a partial R2 domain, we hypothesize that RsMYB1^Short^ may no longer be able to activate the expression of its own encoding gene, which would contribute to the loss of anthocyanin accumulation in white cultivars.

Taken together, our results demonstrate that RsMYB1 was crucial for the accumulation of anthocyanins in radish taproots. However, genotyping of multiple cultivars with the CAPS marker developed in this study also revealed that several radish cultivars with white taproots are heterozygous for the *RsMYB1^Sho^*^rt^ allele. This observation indicates that polymorphisms in other structural and/or regulatory genes contribute to taproot coloration in addition to *RsMYB1*, which is an area for future research. Based on these results, the color of radish roots can be engineered through knowledge-based molecular marker development or manipulation of key genes via genome editing.

## 4. Materials and Methods

### 4.1. Plant Materials

Radish plants were grown in the field under short-day conditions at the National Institute of Agricultural Sciences (Jeonju, Korea). The following six radish cultivars with different root skin colors were used in this study and will be referred to as W1–W3 (for radishes with white taproots) and R1–R3 (for radishes with red taproots): W1, ‘939’ (from Asia seed Co., Seoul, Korea); W2, ‘BaePi’ (from Nongwoo seed Co., Suwon, Korea); W3, ‘948’ (Asia); R1, ‘HongPi’ (Nongwoo); R2, ‘890’ (Asia); and R3, ‘BoraKing’ (Asia). Anthocyanin and the transcript levels of anthocyanin-related genes were analyzed in root skins and root flesh.

For cleaved amplified polymorphic sequences (CAPS) marker analysis, we used a set of 20 radish germplasm obtained from the Agricultural Genetic Resources Center at the National Institute of Agricultural Science (Jeonju, Korea) and from seed companies: the white radishes ‘IT100606’, ‘IT100613’, ‘IT100615’, 939, ‘IT250795’, ‘IT276165’, BaePi, 948 (Asia) and ‘60471’ (Asia); and the red/purple radishes ‘IT261944’, ‘IT2619478’, ‘IT262050’, ‘IT308359’, HongPi, 890, BoraKing, ‘Artesia’ (Asia), and ‘6217’ (Asia).

Transient expression assays with radish cotyledons were conducted using plants of the commercial F_1_ hybrid cultivar ‘DanHong’ (Asia Seed Co.), which were grown in a growth chamber under long day conditions (16 h light/8 h dark) at 22 °C. Tobacco (*Nicotiana tabacum*) plants grown in greenhouses under natural light at 28 °C were used for transient Agrobacterium (*Agrobacterium tumefaciens*)-mediated infiltration assays. All samples were frozen rapidly in liquid nitrogen and stored at –80 °C. A portion of each sample was then used for RNA extraction and anthocyanin measurements.

### 4.2. RNA Extraction, cDNA Synthesis, and Genomic DNA Isolation

Total RNA was extracted from 100 mg of taproots of radish cultivars using the Fruit-mate for RNA Purification solution (Takara, Otsu, Japan) for removal of polysaccharides and polyphenols and Plant RNA Purification Reagent (Invitrogen, Carlsbad, CA, USA) as described previously [39] and purified using the FavorPrep Plant Total RNA Mini Kit (Favorgen, Changzhi, Taiwan). Total RNA was prepared from 100 mg of tobacco leaves using TRIzol reagent (Invitrogen) and purified using the FavorPrep Plant Total RNA Mini Kit (Favorgen), according to the manufacturer’s instructions. DNA contamination was removed by DNase I digestion (Ambion, Thermo Fisher Scientific, MA, USA). First-strand cDNA was synthesized from 2 μg of total RNA using the amfiRivert cDNA Synthesis Platinum Master Mix (GenDEPOT, Barker, TX, USA).

Genomic DNA was extracted from 100 mg of radish leaves using the Plant Mini Kit (Qiagen, Valencia, CA, USA) according to the manufacturer’s instructions.

### 4.3. Measurement of Total Anthocyanin Contents

Total anthocyanin contents in root skins and root flesh were determined according to the method described by [39]. Briefly, samples were ground to powder in liquid nitrogen. Aliquots of 100 mg fresh weight were then mixed in 600 μL extraction buffer (methanol containing 1% (*v*/*v*) HCl) for 6 h at 4 °C with moderate agitation. An addition of 200 μL water and 200 μL chloroform was followed by centrifugation to pellet plant debris at 14,000× *g* for 5 min at 4 °C. After centrifugation, absorbance of the supernatant was recorded at 530 nm (*A*_530_) and 657 nm (*A*_657_) using a microplate reader. Anthocyanin content was determined according to the formula *A*_530_ − (0.33 × *A*_657_). Each sample was extracted and measured from three independent experiments.

### 4.4. RT-qPCR Analysis

Transcript levels were determined by RT-qPCR using the AccuPower 2x Greenstar qPCR Master Mix (Bioneer, Daejun, Korea) and the Bio-Rad CFX96 Detection System (Bio-Rad Laboratories, Hercules, CA, USA) according to the manufacturer’s instructions. The RT-qPCR conditions were used as follows: pre-denaturation at 95 °C for 5 min, 40 cycles of denaturation at 95 °C for 15 s, and annealing at 55 °C for 30 s. Gene expression was normalized using *RNA POLYMERASE II* (*RPII*) and *GLYCERALDEHYDE 3-PHOSPHATE DEHYDROGENASE* (*GAPDH*) for radish and tobacco, respectively, as the reference gene. Three independent biological replicates were performed per sample. The primers used for RT-qPCR analysis are listed in Supplementary Table S1.

### 4.5. Gene Cloning and Sequence Analysis

The full-length open reading frames (ORFs) of *RsMYB1* were amplified from cDNA and genomic DNA derived from white radish (W1) and red radish (R1) cultivars by PCR with PrimeSTAR HS DNA Polymerase (Takara, Japan) using the primer pair RsMYB1 F/R (Supplementary Table S1). The PCR conditions were used as follows: pre-denaturation at 98 °C for 5 min, followed by 35 at 98 °C for 20 s, at 56 °C for 20 s, and at 72 °C for 60 s, and final extension at 72 °C for 5 min. All PCR amplicons were subcloned into the pENTR/D-TOPO vector (Invitrogen) for validation sequencing.

The nucleotide sequences, deduced protein sequences, and ORFs of *RsMYB1* from the W1 and R1 cultivars were analyzed online (http://www.ncbi.nlm.nih.gov, accessed on 13 September 2021). Structural analysis of the deduced proteins was carried out at the ExPASy Molecular Biology Server (http://cn.expasy.org/tools/, accessed on 13 September 2021). Multiple sequence alignments were generated using the CLUSTALW program (https://www.genome.jp/tools-bin/clustalw, accessed on 13 September 2021). A phylogenetic tree was constructed using the neighbor-joining method [40] with MEGA version 6 software [41].

### 4.6. Subcellular Localization Assay

The subcellular localization of RsMYB1^Short^ and RsMYB1^Full^ was analyzed in Arabidopsis protoplasts as described by [42]. Green fluorescent protein (GFP) fusion constructs were generated in the p326-sGFP plasmid, which contains the Cauliflower mosaic virus (CaMV) 35S promoter. For C-terminal GFP fusions, the ORFs of *RsMYB1^Short^* and *RsMYB1^Full^* were individually amplified using gene-specific primer sets (p326-RsMYB1^Short^-F/R and p326-RsMYB1^Full^-F/R), which introduced an *Xba*I restriction site upstream of the ATG codon, using the InFusion Cloning System (Clontech). The resulting p326-RsMYB1^Short^-sGFP and p326-RsMYB1^Full^-sGFP were sequenced to confirm the absence of errors during PCR amplification. The plasmids were introduced into Arabidopsis mesophyll protoplasts by polyethylene-glycol-mediated transformation. After incubation for 16–20 h at 25 °C in the dark, images were captured by fluorescence confocal microscopy (Leica TCS SP8, Leica Microsystems, Germany).

### 4.7. Transactivation and Yeast Two-Hybrid (Y2H) Assays

To generate the *RsMYB1^Short^*, *RsMYB1^Full^*, and *RsTT8* constructs, full-length or truncated versions of the *RsMYB1^Full^* and *RsTT8* ORFs were individually amplified using specific primer sets (Supplementary Table S1). The amplified fragments *RsMYB1^Short^* and *RsMYB1^Full^* were then cloned in-frame with the sequence encoding the GAL4 DNA-binding domain (BD) into pGBKT7 using the In-Fusion Cloning System (Clontech). Similarly, full-length or truncated versions of the *RsTT8* coding sequence were individually cloned in-frame with the sequence of the GAL4 activation domain (AD) into pGADT7. The AD and BD constructs were co-transformed into yeast (*Saccharomyces cerevisiae*) strain MaV203 following the manufacturer’s instructions (Takara). Yeast colonies were selected on synthetic defined (SD) medium lacking Trp and Leu and were replicated on SD medium lacking Trp, Leu, and His containing 10 mM 3-amino-1,2,4-triazole (3-AT), a competitive inhibitor of the *HIS3* gene product. The plates were photographed after 2 d of incubation in the dark at 30 °C.

### 4.8. In Planta Assays of RsMYB1 Function

The plasmid used in tobacco and radish transient expression assays was constructed as follows. The genomic and cDNA sequences of *RsMYB1^Short^* and *RsMYB1^Full^* were amplified with specific primer sets (Supplementary Table S1), and cloned into the pENTR/D-TOPO vector (Invitrogen). For transient assays, the above cloned *RsMYB1^Short^* and *RsMYB1^Full^* PCR amplicons were incorporated into the Gateway destination vector pB7WG2D (VIB-Ghent University, Ghent, Belgium) through several Gateway cloning steps. The resulting vectors were introduced into Agrobacterium strain GV3101 for transient infiltration of the abaxial leaf surface of radish (*Raphanus sativus* cv. ‘DanHong’) and tobacco (*Nicotiana tabacum* cv. Xanthi) plants. Leaf color was monitored 5 d later as described in [27].

### 4.9. Promoter Activation Assay

The *RsCHS* and *RsDFR* promoter regions were individually PCR amplified and then cloned into the pTr-GUS vector to generate reporter constructs as previously described [12]. The ORFs of *RsMYB1^Short^*, *RsMYB1^Full^*, and *RsTT8* were individually subcloned into the pENTR/D-TOPO vector (Invitrogen) and incorporated into the Gateway destination vector pB2GW7 (VIB-Ghent University, Ghent, Belgium) using several Gateway cloning steps. The resulting constructs pB2GW7-RsMYB1^Short^, pB2GW7-RsMYB1^Full^, and pB2GW7-RsTT8 were used as effector constructs. All constructs were transformed into Agrobacterium strain GV3101.

Transient promoter activation assays were performed in tobacco as previously described [12]. Briefly, Agrobacteria containing individual reporter or effector constructs were grown in LB medium for 2 d at 28 °C, pelleted by centrifugation at 3500× *g* for 5 min at 4 °C, resuspended in infiltration buffer (10 mM MgCl_2_ and 100 μM acetosyringone) to an OD_600_ of 0.2 (approximately 10 mL of buffer), and incubated at room temperature without shaking for 2 h. Prior to infiltration into tobacco leaves, Agrobacteria harboring effector and reporter constructs were mixed in a 1:3 ratio. Tobacco leaves were infiltrated with Agrobacteria harboring the genes of interest and harvested to assay GUS activity 3 d later. Agrobacteria harboring only the *GUS* reporter construct were used as controls. At least three independent biological replicates were performed for each experiment.

### 4.10. Molecular Marker for Discrimination of Radish Skin Color

Radish genomic DNA from 20 cultivars (10 white and 10 red/purple) was used to test markers that would discriminate cultivars based on taproot color. PCR was performed with a primer pair for the cleaved amplified polymorphic sequence (CAPS) marker designed against *RsMYB1*. The PCR conditions were as follows: denaturation at 98 °C for 2 min, followed by 30 cycles of 98 °C for 10 s, 60 °C for 15 s, and 68 °C for 30 s. For CAPS analysis, PCR amplicons were digested with *Mlu*CI, separated on a 1.5% agarose gel, and visualized by staining with ethidium bromide.

## 5. Conclusions

In this study, we demonstrated that anthocyanin biosynthesis is determined in part by allelic variation at the *RsMYB1* locus in the form of the *RsMYB1^Short^* and *RsMYB1^Full^* alleles. Sequence analysis revealed a 4 bp insertion in the white allele *RsMYB1^Short^* in the first exon, leading to a frameshift mutation that produced the truncated protein with a partial R2 domain. In radish cultivars with red taproots, anthocyanin accumulation and anthocyanin biosynthetic gene expression strongly depended on the transcript levels of *RsMYB1^Full^* and *RsTT8*. A transient expression assay with radish cotyledons indicated that *RsMYB1^Short^* may encode a non-functional protein that fails to induce the accumulation of anthocyanins. Promoter activation assays and transient expression assays in tobacco demonstrated that co-expressing *RsMYB1^Full^* and *RsTT8* activated anthocyanin accumulation and *RsCHS* and *RsDFR* transcription, whereas co-expressing *RsMYB1^Short^* and *RsTT8* did not. Taken together, these results suggest that the *RsMYB1* allele plays a key role in shaping anthocyanin accumulation in the radish taproot.

## Data Availability

Not applicable.

## Supplementary Materials

The following are available online at https://www.mdpi.com/article/10.3390/ijms222010927/s1. Figure S1: Multiple alignment of the genomic sequence of RsMYB1 derived from red (RsMYB1^Full^) and white (RsMYB1^Short^) radish and the previously reported RsMYB1 genomic sequence from the Bordeaux cultivar, Figure S2: Phylogenetic relationships between anthocyanin biosynthetic regulators in radish and other species, Table S1: List of primers used in this study.
