# Supplementary material for: Loss of the R2R3 MYB Transcription Factor RsMYB1 Shapes Anthocyanin Biosynthesis and Accumulation in Raphanus sativus
Source: Int J Mol Sci. 2021 Oct 10;22(20):10927. doi: 10.3390/ijms222010927 (PMC8535906; doi:10.3390/ijms222010927)
Supplement: Supplementary file 1 [file ijms-22-10927-s001.zip › Supplementary Table (RsMYB1)_1003_revised.pdf]

Supplementary Table S1. List of primers used in this study.

Usage	Primer name	Primer sequence (5'-3')	Amplicon size
Gene expression	qRT-RsPAL-F	CGTCTCCTCAGTGGCTAG	126 bp
	qRT-RsPAL-R	CGTGAATCGCTTTGTTCCT	
	qRT-RsCHS-F	GTGACTGGAACTCCCTCT	99 bp
	qRT-RsCHS-R	CTCTCATCTTCTCAGCCTTG	
	qRT-RsCHI-F	TCCATCCTCTTCGCTCTC	149 bp
	qRT-RsCHI-R	GACACACGGTTCTTTCCAA	
	qRT-RsF3H-F	TTACAAGCCACACGAGAC	84 bp
	qRT-RsF3H-R	ATGGTCGCCTAGATTAACAAC	
	qRT-RsDFR-F	CGTTAGCGGAGAAAGCAG	109 bp
	qRT-RsDFR-R	GGCGGCATAGATGTTGTTAT	
	qRT-RsANS-F	GAAGTTGGTGGCTTAGAAGAG	134 bp
	qRT-RsANS-R	ATGTTGTGTAGAATCAAGGTCAA	
	qRT-RsMYB1-F	GTGCATGGACTGCTGAAGAA	258 bp
	qRT-RsMYB1-R	CAGTCCGACCGGGTAATCTA	
	qRT-RsTT8-F	AGTGATCGGAGCTGAGGAAA	393 bp
	qRT-RsTT8-R	ACTTGCTTCCTCCTCGCATA	
	qRT-RsTTG1-F	AACAGCAAGACGTCCGAGTT	171 bp
	qRT-RsTTG1-R	GATGTCGTGGACCTCCTTGT	
	qRT-RsRPII-F	ATCACGCTAAATGGTCTCCT	122 bp
	qRT-RsRPII-R	GCTGCTCTCAATCAAGTCAATC	
	qRT-NtPAL-F	ATTGAGGTCATCCGTTCTGC	102 bp
	qRT-NtPAL-R	ACCGTGTAACGCCTTGTTTC	
	qRT-Nt4CL-F	TCATTGACGAGGATGACGAG	114 bp
	qRT-Nt4CL-R	TGGGATGGTTGAGAAGAAGG	
	qRT-NtCHS-F	TTGTTCGAGCTTGTCTCTGC	116 bp
	qRT-NtCHS-R	AGCCCAGGAACATCTTTGAG	

	qRT-NtCHI-F	GTCAGGCCATTGAAAAGCTC	103 bp
	qRT-NtCHI-R	CTAATCGTCAATGCCCCAAC	
	qRT-NtF3H-F	CAAGGCATGTGTGGATATGG	92 bp
	qRT-NtF3H-R	TGTGTCGTTTCAGTCCAAGG	
	qRT-NtFLS-F	TTTGGCACTTGGTGTTGTGG	113 bp
	qRT-NtFLS-R	ACTTGACATCATACCAATGG	
	qRT-NtF3'H-F	AGGCTCAACACTTCTCGT	119 bp
	qRT-NtF3'H-R	CATCAACTTTGGGCTTCT	
	qRT-NtDFR-F	AACCAACAGTCAGGGGAATG	104 bp
	qRT-NtDFR-R	TTGGACATCGACAGTTCCAG	
	qRT-NtANS-F	TGGCGTTGAAGCTCATACTG	118 bp
	qRT-NtANS-R	GGAATTAGGCACACACTTTGC	
	qRT-NtUFGT-F	CAATGTTTGGGATGGTGTCA	124 bp
	qRT-NtUFGT-R	TTCCTCCTCTGCCTCTTTCA	
	qRT-NtGAPDH-F	GGTGTCCACAGACTTCGTGG	220 bp
	qRT-NtGAPDH-R	GACTCCTCACAGCAGCACCA	
Gene cloning	$RsMYB1^{Full}$-F	ATGGAGGGTTCGTCCAAAGGG CTGAG	747 bp
	$RsMYB1^{Full}$-R	TTACACAGTCTCTCCATCTAACAGGCT	
	$RsMYB1^{Short}$-F	ATGGAGGGTTCGTCCAAAGGG CTGAG	751 bp
	$RsMYB1^{Short}$-R	TTACACAGTCTCTCCATCTAACAGGCT	
Subcellular localization	p326-$RsMYB1^{Full}$-F	CACGGGGGACTCTAGAATGGAGGGTTCGTCCAAAG	776 bp
	p326-$RsMYB1^{Full}$-R	CCATGGATCCTCTAGACACAGTCTCTCCATCTAAC	
	p326-$RsMYB1^{Short}$-F	CACGGGGGACTCTAGAATGGAGGGTTCGTCCAAAG	155 bp
	p326-$RsMYB1^{Short}$-R	CCATGGATCCTCTAGAGCTCTTAAAGGAATTAACTTGG	
Yeast two hybrid	pGADT7-$RsMYB1^{Full}$-F	GGAGGCCAGTGAATTCATGGAGGGTTCGTCCAAAGGG	779 bp
	pGADT7-$RsMYB1^{Full}$-R	CACCCGGGTGGAATTCTTACACAGTCTCTCCATCTAACAGG	
	pGADT7-$RsMYB1^{Full}$ N1-F	GGAGGCCAGTGAATTCATGGAGGGTTCGTCCAAAGGG	224 bp
	pGADT7-$RsMYB1^{Full}$ N1-R	CACCCGGGTGGAATTCCCCTCTCTTGATACTTGGC	

	pGADT7-RsMYB1^{Full} N2-F	GGAGGCCAGTGAATTCTTGAACTATTTGAAGCCAA	236 bp
	pGADT7-RsMYB1^{Full} N2-R	CACCCGGGTGGAATTCCCCTCTCTTGATACTTGGC	
	pGADT7-RsMYB1^{Full} N3-F	GGAGGCCAGTGAATTCATGGAGGGTTCGTCCAAAGGG	395 bp
	pGADT7-RsMYB1^{Full} N3-R	CACCCGGGTGGAATTCCCCTCTCTTGATACTTGGC	
	pGADT7-RsMYB1^{Full} C-F	GGAGGCCAGTGAATTCGAACCAGGTTGTAAGACAC	443 bp
	pGADT7-RsMYB1^{Full} C-R	CACCCGGGTGGAATTCTTACACAGTCTCTCCATCTAACAGG	
	pGADT7-RsMYB1^{Short}-F	GGAGGCCAGTGAATTCATGGAGGGTTCGTCCAAAGGG	153 bp
	pGADT7-RsMYB1^{Short}-R	CACCCGGGTGGAATTCGCTCTTAAAGGAATTAACTTGG	
	pGBKT7-RsTT8-F	CATGGAGGCCGAATTCATGGATGAATCAAGTATTATAC	1589 bp
	pGBKT7-RsTT8-R	GGATCCCCGGGAATTCGAGTTTATTTTGAGATAT	
	pGBKT7-RsTT8M-F	CATGGAGGCCGAATTCATGGATGAATCAAGTATTATAC	635 bp
	pGBKT7-RsTT8M-R	GGATCCCCGGGAATTCGAAGAAACTCTTCATGTGTTCAAC	
	pGBKT7-RsTT8N-F	CATGGAGGCCGAATTCATGGATGAATCAAGTATTATAC	1076 bp
	pGBKT7-RsTT8N-R	GGATCCCCGGGAATTCAGGAACTCTCAAGATCATGTGTTTG	
	pGBKT7-RsTT8C-F	CATGGAGGCCGAATTCCATGAAGAAGACGAAGAAGTAG	926 bp
	pGBKT7-RsTT8C-R	GGATCCCCGGGAATTCGAGTTTATTTTGAGATAT	
In planta assay	RsMYB1^{Full}-F	ATGGAGGGTTCGTCCAAAGGGCTGAG	747 bp
	RsMYB1^{Full}-R	TTACACAGTCTCTCCATCTAACAGGCT	
	RsMYB1^{Short}-F	ATGGAGGGTTCGTCCAAAGGGCTGAG	126 bp
	RsMYB1^{Short}-R	TCAGCTCTTAAAGGAATTAACTTGGT	
	gRsMYB1^{Full}-F	ATGGAGGGTTCGTCCAAAGGGCTGAG	1250 bp
	gRsMYB1^{Full}-R	TTACACAGTCTCTCCATCTAACAGGCT	
	gRsMYB1^{Short}-F	ATGGAGGGTTCGTCCAAAGGGCTGAG	1287 bp
	gRsMYB1^{Short}-R	TTACACAGTCTCTCCATCTAACAGGCT	
	pB7WG2D-RsMYB1^{Full}-F	AAAAAAGCAGGCTTTATGGAGGGTTCGTCC	770 bp
	pB7WG2D-RsMYB1^{Full}-R	GTACAAGAAAGCTGGGTCTTACACAGTCTCTCCA	
	pB7WG2D-RsMYB1^{Short}-F	AAAAAAGCAGGCTTTATGGAGGGTTCGTCC	159 bp
	pB7WG2D-RsMYB1^{Short}-R	GTACAAGAAAGCTGGGTCTTACACAGTCTCTCCA	

	pB7WG2D-gRsMYB1^{Full}-F	AAAAAAGCAGGCTTTATGGAGGGTTCGTCC	1283 bp
	pB7WG2D-gRsMYB1 ^{Full}-R	GTACAAGAAAGCTGGGTCTTACACAGTCTCTCCA	
	pB7WG2D-gRsMYB1^{Short}-F	AAAAAAGCAGGCTTTATGGAGGGTTCGTCC	1320 bp
	pB7WG2D-gRsMYB1^{Short}-R	GTACAAGAAAGCTGGGTCTTACACAGTCTCTCCA	
Molecular marker	CAPS-RsMYB1^{Full}-F	CAAGCCTGCCTTTACGTATACTTAAAA	216 bp
	CAPS-RsMYB1^{Full}-R	TGTGCAAAATGATAAAAAGTAACATACC	
	CAPS-RsMYB1^{Short}-F	CAAGCCTGCCTTTACGTATACTTAAAA	220 bp
	CAPS-RsMYB1^{Short}-R	TGTGCAAAATGATAAAAAGTAACATACC	

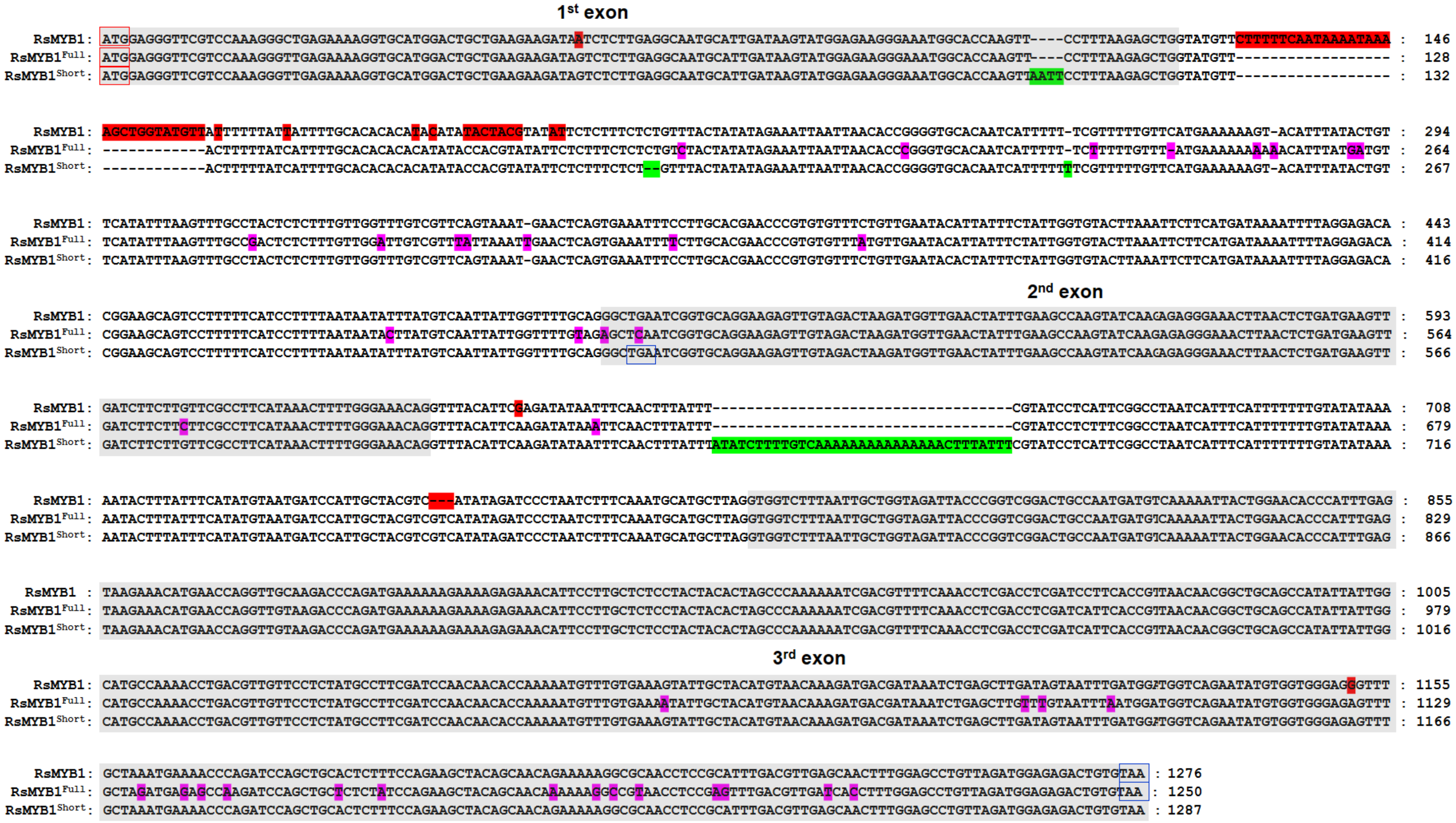

Supplementary Figure S1. Multiple alignment of the genomic sequence of *RsMYB1* derived from red (*RsMYB1^{Full}*) and white (*RsMYB1^{Short}*)

radish and the previously reported *RsMYB1* genomic sequence from the Bordeaux cultivar. The start and stop codons are indicated by a red and blue box, respectively. Exons are indicated by gray boxes. Single-nucleotide polymorphisms (SNPs) and insertions and/or deletions (Indels) from *RsMYB1*, *RsMYB1*^{Full} and *RsMYB1*^{Short} are highlighted in red, pink and green color, respectively.

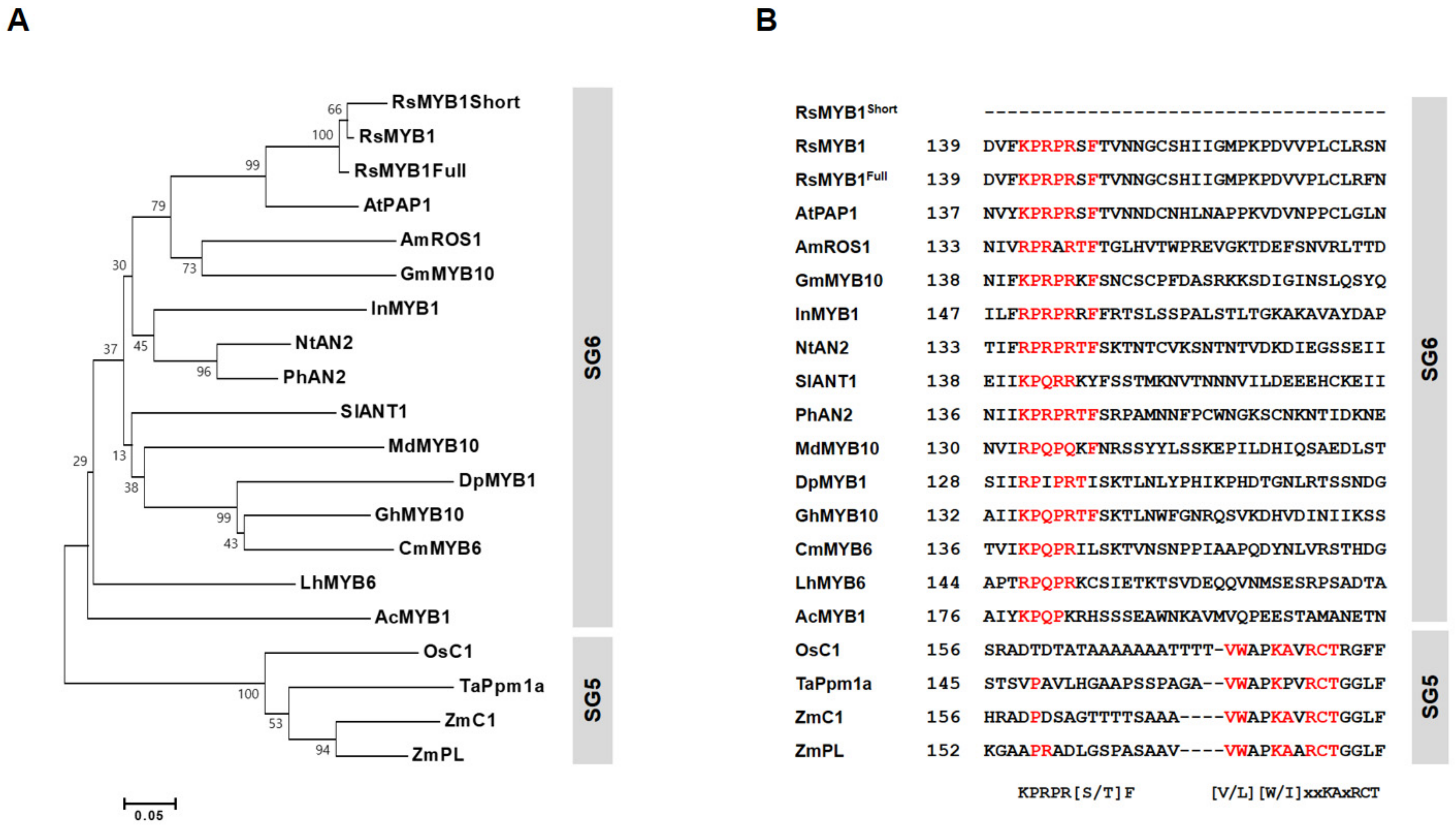

Supplementary Figure S2. Phylogenetic relationships between anthocyanin biosynthetic regulators in radish and other species. (A)

Phylogenetic tree of radish RsMYB1 and R2R3 MYB proteins from other plants. The phylogenetic tree was constructed using the neighbor-joining method with MEGA6 software. Anthocyanin-activating R2R3-MYBs are divided into subgroup 5 (SG5) and SG6, based on the conserved SG5 and SG6 motifs, respectively. GenBank accession numbers are *Allium cepa* AcMYB1 (KX785130); *Antirrhinum majus* AmROS1 (ABB83826); *Arabidopsis thaliana* AtPAP1 (AAG42001); *Chrysanthemum x morifolium* CmMYB6 (AKP06190); *Dahlia pinnata* (syn. *variabilis*) DpMYB1 (AB601003); *Garcinia mangostana* GmMYB10 (ACM62751); *Gerbera hybrida* GhMYB10 (CAD87010); *Ipomoea nil* InMYB1 (BAE94391); *Lilium hybrida* LhMYB6 (BAJ05399); *Malus × domestica* MdMYB10 (ACQ45201); *Nicotiana tabacum* NtAN2 (ACO52472); *Oryza sativa* OsC1 (BAD04024); *Petunia × hybrida* PhAN2 (AAF66727); *Raphanus sativus* (KR706195); *Solanum lycopersicum* SlANT1 (AAQ55181); *Triticum aestivum* TaPpm1 (MG066451); *Zea mays* ZmC1 (AAA33482), ZmPL (AAA19819).(B) Multiple protein sequence alignment of a part of the C-terminal region of R2R3-MYBs, focusing on the SG5 and SG6 motifs shown in (A).
